# Dr. Jia-Xiang Shen: a pioneer of the Chinese pharmaceutical industry

**DOI:** 10.1007/s13238-016-0294-1

**Published:** 2016-07-18

**Authors:** Xianghai Guo, Baozhi Han

**Affiliations:** 1 Department of Pharmaceutical Engineering, School of Chemical Engineering and Technology, Tianjin University, Tianjin, 300350 China; 2 Key Laboratory of System Bioengineering, Ministry of Education, Tianjin, 300350 China; 3 Archives Department, Tianjin University, Tianjin, 300072 China

In 2007, Dr. Jia-Xiang Shen received the “Outstanding Contribution Award” at the centenary celebrations of Chinese Pharmaceutical Association. Dr. Shen was acknowledged for his study on the new methodology for the total synthesis of chloramphenicol, the industrialization of several important steroid hormone drugs, and for being a co-founder of Chinese modern pharmaceutical industry.

Dr. Shen was born on November 11, 1921 in Yangzhou, Jiangsu Province, and later on moved to Nanjing with his parents. As a teenager, he studied at Nanjing Municipal High School where he was well trained. When the War of Resistance against Japanese Aggression broke out in 1937, the school was suspended. To make things worse, Dr. Shen got infected with tuberculosis, so he had no choice but moving to Chongqing with his family. One year later, with excellent exam scores, Dr. Shen was admitted to the National Advanced Pharmacy College (the predecessor of China Pharmaceutical University), which was just moved from Nanjing to Chongqing. During this time, Dr. Shen became extremely interested in Medicinal Chemistry with the influence of Prof. Xinghan Lei. This period of educational experiences, though turbulent but self-disciplined, laid foundation for his life-long path on pharmacy.

After graduation, Dr. Shen worked shortly in the Army Pharmaceutical Institute. Then, he went to England to study at the School of Pharmacy in University of London, U.K (Fig. 1). It took him only four years to complete all the courses required for both bachelor and doctoral degrees. Dr. Shen received his PhD degree in Medicinal Chemistry from University of London in 1949 (Linnell and Shen, 1949; Linnell and Shen, 1951). Immediately after graduation, he made up his mind to return back to China. He was so eager to devote himself to the development of the newly founded People’s Republic of China that he couldn’t even wait to receive the award of his PhD certificate. On September 23, 1949, Dr. Shen boarded on a ship and began the voyage back to his homeland via Hong Kong. He became one of the first Western-trained Chinese scholars returning back to China. Since then, Dr. Shen has dedicated his entire life to the development of pharmaceutical industry in China and is renowned as one of the founding members of pharmaceutical industry.

**Figure 1:**
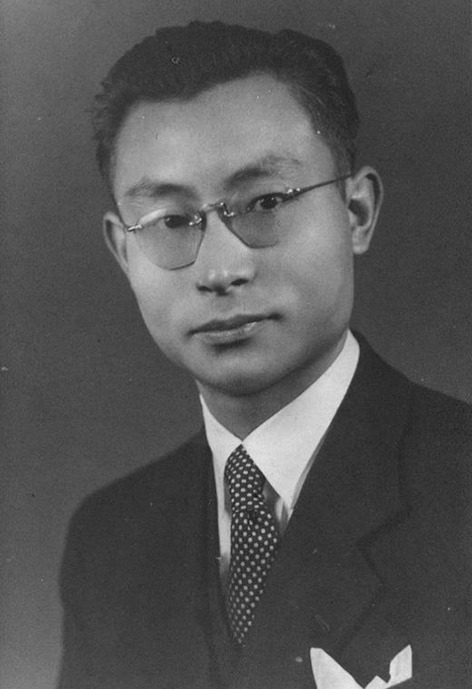
Jia-Xiang Shen at age 24, studying at University of London (1945)

During the Korean War, Dr. Shen took the task to lead the development of chloramphenicol, a badly-needed antibiotic for wounded Chinese soldiers. Under his guidance, a new synthetic method of chloramphenicol was developed. Within a few years, he achieved many significant technical innovations during the production processes, which significantly improved the manufacturing technique of chloramphenicol (Fig. 2). In 1957, the new technique was successfully applied into large-scale production, which is hailed as an important milestone marking the beginning of modern pharmaceutical industry in China (Shen et al., 1950; Shen et al., 1958a, b, c, d, e, f). In the 1950s and 1960s, he successfully synthesized crystalline Vitamin A acetate and Vitamin D_2_ using domestic resources, supervised the synthesis and production of multiple steroid medicines such as hydrocortisone and dexamethasone (Shen et al., 1964a, b, c), and accomplished the total synthesis of gestrinone, which laid a solid foundation for the industrial synthesis of 19-demethyl steroid drugs in China.

**Figure 2:**
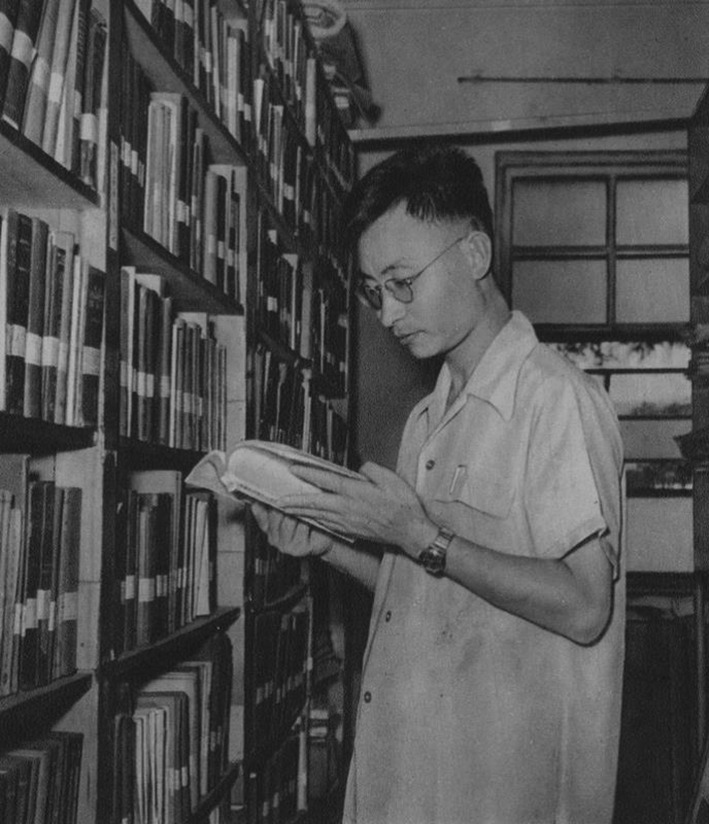
Jia-Xiang Shen was searching documents for chloramphenicol at a library (1957)

Towards the end of “Cultural Revolution” in 1973, Dr. Shen was reappointed as the Deputy Chief Engineer of Hunan Pharmaceutical Industry Research Institute. Shortly after that, he embarked on a new research topic based on effective constituents of Chinese Traditional Medicines (CTM). He demonstrated the unique chemical structure of agrimophol through the total synthesis method in 1976 (Shen et al., 1976a, b), which was the active ingredient of an anti-tapeworm drug. In mid-1980s, he was appointed as the doctoral advisor in medicinal chemistry at Beijing Medical University (now part of Peking University) (Fig. 3). He supervised the PhD candidates on the study of the total synthesis of *Tanshinone IIA* and *Danshenxinkun B* (1986–1988) (Shen et al., 1988; Zhang and Shen, 1988), and discovered the special pharmacological activities in some derivatives. In addition, his group also studied the methodology of total synthesis of *erycibe alkaloid II* (baogongteng A) and the analogues.

**Figure 3:**
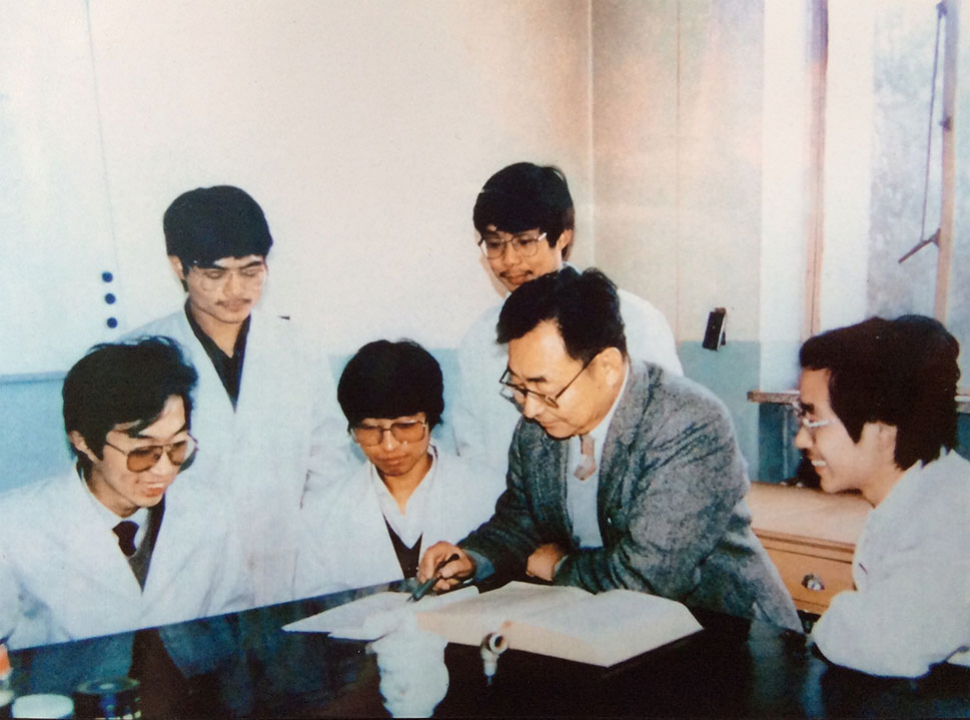
Dr. Shen was advising his graduate students at Beijing Medical University (now part of Peking University) (1987)

In late 1992, after his retirement, as the Deputy Chief Engineer of the State Pharmaceutical Administration of China and the Director General of the National Institutes of Pharmaceutical R&D, Dr. Shen established Beijing Jicai Pharmaceutical Research Institute. It was the first private pharmaceutical research institute in China, where he discovered a new crystal form of azithromycin. This discovery bypassed Pfizer’s administrative restriction for azithromycin dehydrate in China. Later on, this product occupied the majority of domestic market share of Azithromycin with competitive price advantages, which substantially improved the access to the Chinese people. Additionally, his laboratory also overcame various technical challenges and developed for the first time in China various hard-to-synthesize specialty generics such as alfacalcidol, tibolone, budesonide, and tamsulosin.

With the development of China’s science and technology, Dr. Shen became increasingly recognized in the international pharmaceutical field (Shen and Zhuang, 1984). Given his distinguished contributions and international reputation in the field of medicinal chemistry, Dr. Shen was elected as the Communication Academician of France Medication Academy in 1983. In the 1980s, Dr. Shen attended many conferences held by the United Nations Industrial Development Organization and the World Health Organization. In 1987, he was appointed as a member of the World Health Organization’s leading group working on the chemotherapy for the treatment of Malaria and played a critical role in introducing the anti-malarial medicine artemisinine which was developed in China to the world (Luo and Shen, 1987; Shen, 1991). In 1988, he held the Directorship of Sino-Searle Foundation. In 1989, he was invited to join the editorial board of *Medicinal Research Reviews* and the *Journal of Pharmaceutical Sciences*.

Dr. Shen took the development of pharmaceutical science, the promotion of China’s pharmaceutical industry and the health of people as his own duty, and went through an extraordinary way along the stormy development path of Chinese pharmaceutical industry. He advanced the basic research and key technologies of Chinese pharmaceutical science, advocated the translation and application of research achievements. With his distinguished achievements, Dr. Shen won two prizes of National New Product Award (1964), five prizes of National Scientific Conference Award (1978), the Third Class of National Invention Award (1982), and the Second Class for Beijing Science and Technology Progress Award (1999). In 1999, he was elected as a member of the prestigious Chinese Academy of Engineering.

Dr. Shen witnessed and experienced China’s poverty during his youth, and he felt deeply humiliated by the aggressions of foreign powers. This inspired him to work hard for China’s rejuvenation and empower the nation with modern science. He summarized his research style as “subject driven by mission” and “starting with easy things, but never leave without digging in great depth”. He always chose the research topics that are valuable to the development and progress of the country. He told his students many times, “As explorers of science, we must uphold the rule of self-reliance. Contributing to your country, rather than living on its support.”

In 2001, at the age of 80, Dr. Shen gave up his quiet and comfortable life in Beijing and accepted the invitation to join Tianjin University with the hope of advancing pharmaceutical science and industry through education. He moved to Tianjin and co-founded the School of Pharmaceutical Science and Technology together with other colleagues at Tianjin University. During the school’s early days, he always worked in the frontline regardless of his age and health. Everyone at Tianjin University was deeply inspired by his persevering spirit, which has become invaluable wealth to this institution.

## References

[CR1] Linnell WH, Shen CC (1949). Synthesis of the benzene analogues of vitamin A. J Pharm Pharmacol.

[CR2] Linnell WH, Shen CC (1951). Rupe’s rearrangement. J Pharm Pharmacol.

[CR3] Luo X-D, Shen J-X (1987). the chemistry, pharmacology and clinical applications of qinghaosu (artemisinin) and Its derivatives. Med Res Rev.

[CR4] Shen J-X (1991). Antimalarial drug development in China.

[CR5] Shen J-X, Zhuang l-G (1984). Current trends in new drug research in People’s Republic of China. Med Res Rev.

[CR6] Shen J-X, Guo K-Y, Gao P-M (1950). Synthesis of chloramphenicol (I). Chin Sci Bull.

[CR7] Shen J-X, Zhang Y-Q, Zhou B-W (1958). Synthesis research of chloramphenicol II–VII. Acta Pharm Sin.

[CR8] Shen J-X, Guan J-H, Yang Q-T (1958). Synthesis research of chloramphenicol (III). New synthesis method of P-nitroacetophenone by the auto-oxidation of nitroethylbenzene. Acta Pharm Sin.

[CR9] Shen J-X, Zhou B-W, Pan F-P (1958). Synthesis research of chloramphenicol (IV). Research and improvement of epichlorohydrin aluminum reduction. Acta Pharm Sin.

[CR10] Shen J-X, Xie K, Cai Y-Z (1958). Synthesis research of chloramphenicol (V). Partition of DL-threo-1-parachloronitrobenzene-2-amino-1,3-propylene glycol. Acta Pharm Sin.

[CR11] Shen J-X, Wang Q-F, Cai Y-Z (1958). Synthesis research of chloramphenicol (VI). Racemization and reduction of l-α-dichloro acetyl-β-hydroxy-nitrobenzene acetone. Acta Pharm Sin.

[CR12] Shen J-X, Cai Y-Z, Pan F-P (1958). Synthesis research of chloramphenicol (VII). Synthesis research ofmethyl dichloroacetate. Acta Pharm Sin.

[CR13] Shen J-X, Li T-S, Wang Q-F (1964). Steroid hormone. Acta Pharm Sin.

[CR14] Shen J-X, Wang Q-F, Cai Y-K (1964). Steroid hormone II. Acta Pharm Sin.

[CR15] Shen J-X, Chen Y-Y, Zhang X-D (1964). Steroid hormone III. Acta Pharm Sin.

[CR16] Shen J-X, Ning D-Z, Zhang L-Y (1976). Complete synthesis of agrimophol. Chin Herb Med Commun.

[CR17] Shen J-X, Ning D-Z, Zhang L-Y (1976). Complete synthesis of agrimophol. Acta Chim Sin.

[CR18] Shen J-X, Zhang P-Z, Qiao M (1988). New complete synthesis method of tanshinone IIA, the effective constituent in CTM salvia. Acta Pharm Sin.

[CR19] Zhang P-Z, Shen J-X (1988) New complete synthesis of Danshenxinkun B, the effective constituent in CTM salvia. In: Proceedings of the third national conference of natural pharmaceutical chemistry (Shanghai)

